# The UK kidney association 2025 academic census: a national survey to identify workforce improvements

**DOI:** 10.1186/s12909-026-08855-y

**Published:** 2026-02-20

**Authors:** Louise Oni, Alice Saunders, Kelly Vernon, Hayley Hardwick, John A Sayer, Kathrine Parker

**Affiliations:** 1 Department of Women’s and Children’s Health, Institute of Life Course and Medical Sciences, University of Liverpool, Liverpool, UK; 2 Department of Paediatric Nephrology, Alder Hey Children’s NHS Foundation Trust Hospital, Liverpool, UK; 3 UCL Centre for Kidney and Bladder Health, UCL, London, UK; 4 North West School of Paediatrics, NHS Health Education England, Liverpool, UK; 5 Biosciences Institute, Faculty of Medical Sciences, Newcastle University, Central Parkway, Newcastle upon Tyne, NE1 3BZ UK; 6 Renal Services, Newcastle upon Tyne NHS Foundation Trust, Newcastle upon Tyne, UK; 7 Newcastle Biomedical Research Centre, National Institute for Health Research, Newcastle Upon Tyne, UK; 8 Institute of Nephrology and Transplantation, Manchester University NHS Foundation Trust, Oxford Road, Manchester, UK; 9 Department of Pharmacy and Optometry, University of Manchester, Stopford Building, Oxford Road, Manchester, UK

**Keywords:** Academic workforce, Nephrology research, Career barriers, Workforce development, UK renal medicine

## Abstract

**Background:**

Academic nephrology underpins innovation, translation, and the training of the future kidney workforce, yet there is growing concern that its capacity in the UK is fragile. Increasing clinical service pressures, limited protected academic time, fragmented career pathways, and challenges in recruitment and retention threaten the sustainability of the academic kidney workforce, prompting the UK Kidney Association (UKKA) to undertake a national Academic Census to systematically define these barriers and identify priorities for workforce improvement.

**Methods:**

The anonymous survey was co-designed by UKKA and British Association for Paediatric Nephrology (BAPN), drawing on established academic workforce survey instruments and distributed nationally through UKKA and BAPN communication channels and their respective membership networks targeting health care professional, clinician scientists and basic scientists who spent the majority (> 50%) of their working time in Renal Medicine.

**Results:**

A total of 225 responses were recorded, of which 30.4% were over the age of 50 years, 32.6% were age 41–50 years and 28.6% age 31–40 years. Just 8.4% of respondents were under the age of 30 years. Half of respondents were medical doctors, 10% were nurses, 21% were allied health professionals and 18.7% were scientists. Of the cohort, 25.8% (*n* = 58) worked in paediatric medicine. Over half (52%, *n* = 118) had a PhD or MD, and 41.8% (*n* = 94) had over 20 years’ professional experience. The age distribution and experience indicated that most respondents were in the latter stage of their careers. The survey established barriers preventing successful academic careers and these included a lack of time (64%, *n* = 145), lack of research funding (60%, *n* = 135) and competing work roles (53%, *n* = 120).

**Conclusions:**

This academic survey provides insight into how to strengthen the academic workforce for nephrology research in the UK.

**Supplementary Information:**

The online version contains supplementary material available at 10.1186/s12909-026-08855-y.

## Background

 Kidney disease represents an under-recognised global challenge with rising rates of chronic kidney disease (CKD) and kidney failure placing a high demand on healthcare services. Changing the trajectory of the CKD and kidney failure crisis requires an active effort to incorporate research into clinical care to bring rapid advances to patients living with this damaged vital organ. However, there is a national crisis in clinical academic capacity across the UK, and internationally, as outlined by the Medical Research Council and Baroness Brown of Cambridge in her review of Clinical Academics in the NHS, 2023 [1, 2]. A major concern is that there remains substantially fewer younger clinical academics under the age of forty years to replace those who will retire in the next ten years, and as such the situation requires critical evaluation.

The workforce crisis is most apparent in subspeciality areas and underrepresented groups such as paediatrics. National initiatives have recognised the crisis, acknowledging the vital benefits that medical and scientific research brings to healthcare and to improving patient outcomes [3]. In the UK, despite the previous introduction of the academic clinical training pathway through the National Institute of Health Research (NIHR), which funded 278 Academic Clinical Fellowships in 2024/25, the crisis remains, with only 50% of doctors upon completion of the academic training pathway successfully securing a clinical academic post [4–6]. There is also an underrepresentation of allied healthcare professionals (AHPs) in research to develop representative portfolios of research. The barriers to research have been widely reported [7–11], with many proposals on how to improve the research landscape, however only one previous study has looked specifically into early-career clinicians contributing to nephrology research in the UK [12], despite this being a high clinically demanding speciality [13].

Using a pan-specialty approach, colleagues affiliated with advancing paediatric and adult nephrology research under the oversight of the UK Kidney Association (UKKA), were keen to inform a comprehensive research strategy building on previous annual reviews. To understand the current research environment, the UKKA 2025 academic census was designed with the aim to capture workforce feedback to guide a strategy for improvement and advocate for kidney disease research on a national basis.

## Methods

The anonymous survey was developed through a collaborative process led by the UKKA in partnership with the British Association for Paediatric Nephrology (BAPN), informed by review of previously published national academic workforce surveys and adapted to reflect the UK nephrology context. The survey was distributed nationally through UKKA and BAPN communication channels and their respective membership networks targeting health care professional, clinician scientists and/or basic scientists targeting those who spend the majority (> 50%) of their working time in the kidney field. The survey was distributed via the UKKA and British Association of Paediatric Nephrology e-newsletters, Kidney Research UK e-mail distribution lists and via direct emails to leading academic institutions. Reminder emails were sent via the UKKA fortnightly over an 8 week period. The survey was hosted on a Google survey form (see Supplementary data) and the survey was open for a period of 8 weeks from 1st January until 28th February 2025.

The questions asked included; age in ranges, ethnicity, professional background, job title, location, current employer, affiliated clinical and/or academic centre, setting, number of professional years’ experience, highest level of qualifications, whether research related activities were part of current or previous roles, time spent on research related activity, time formally recognised for research related activity, research activity discussed in annual appraisal, engagement in research, research activities in past 12 months, rating of current success or skill level in certain areas, barriers and motivators to research, top three personal barriers, mentorship, and organisational support. All respondents were offered the opportunity to be contacted with meta-data.

Data was analysed and represented in graphical form using Microsoft Excel. Respondents were divided into broad groups based on their professional background: doctors, scientists, and allied health professionals (AHP) which included nurses, dieticians, pharmacists, occupational therapists, psychologists, counsellors, and social workers. Subgroup analysis was performed for respondents working solely in paediatrics.

No ethical approval was required for this study as this study involved voluntary data collected from a professional survey whereby respondents were informed prior to completing the survey that the results would be used to inform future improvements by analysing current engagement with nephrology research. Consent was assumed by the completed of the survey, and the data was anonymised for analysis.

## Results

### Demographics and experience of the respondents

**Fig. 1:**
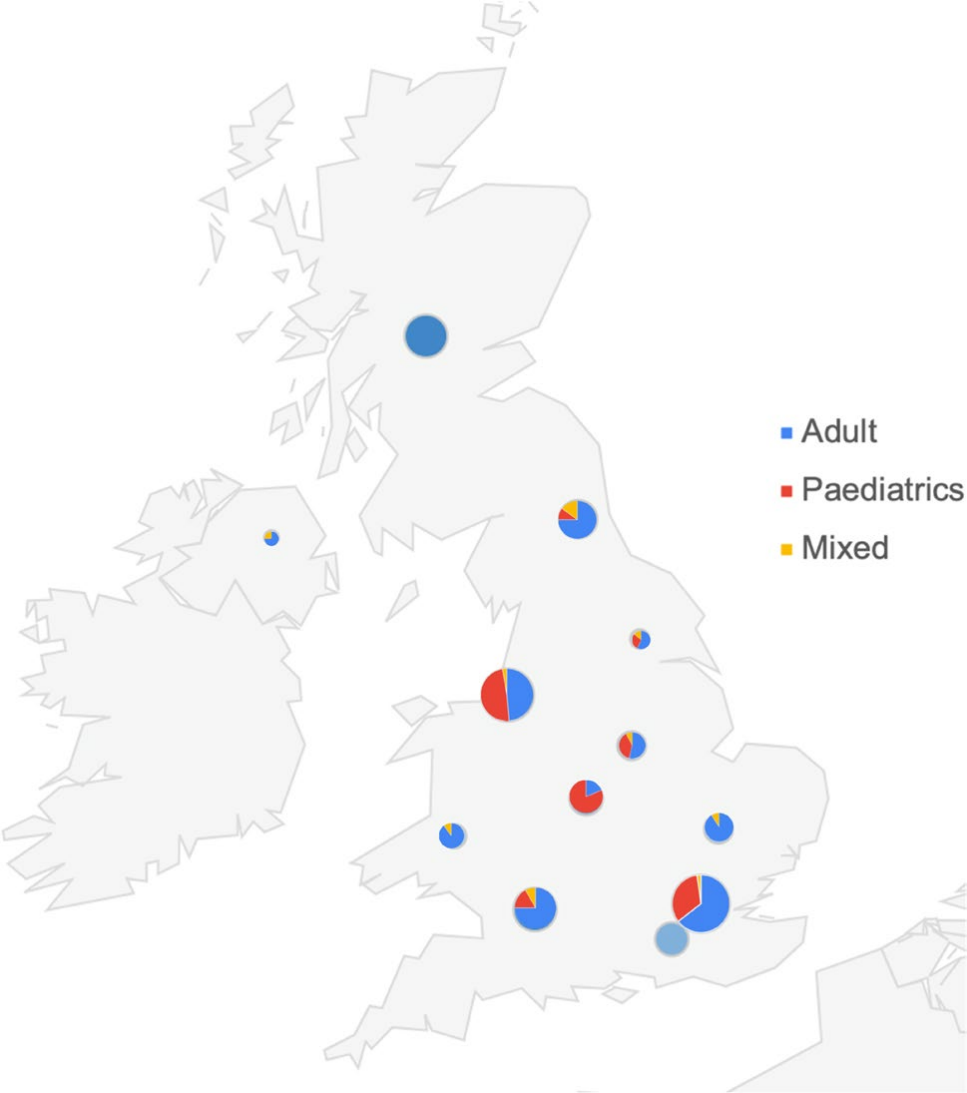
Geographical distribution of respondents. Map of UK with circles drawn to scale to represent the number of responses. Pie charts represent distribution of adult, paediatric or mixed roles

The open distribution of the survey precluded calculation of a formal response rate, which represents a limitation of the study. Overall, 225 responses were recorded (Fig. 1). Incomplete survey responses were included and analyses were performed using available-case data.

The survey received 225 responses, with a bias towards a senior, experienced workforce: 63% were over 40 years old and 41.8% had over 20 years of professional experience. The cohort was predominantly White British (70.5%) and worked in secondary or tertiary care (75%). Medical doctors comprised half the respondents (50%), followed by nurses/AHPs (31.5%) and scientists (18.7%), with many holding senior academic positions like professor or principal investigator roles. Over half of all respondents (52%) held a PhD or MD as their highest qualification. Research activities were part of the current role for 81.4%, though nearly half (49.4%) spent less than 25% of their time on it. The main motivators were to inform clinical practice (68.9%) and improve services (62.7%), while lack of dedicated time (64.4%) and funding (60%) were the primary barriers. A subgroup analysis of the paediatric workforce demonstrated that they had a fewer people with a PhD or MD as their highest level of qualification and less dedicated time spent on research, otherwise the findings were like the whole cohort as summarised in Table 1.

**Table 1:** A summary of the responses from the paediatric academic workforce compared to the whole cohort or respondents involved in research for both adults and children

	Whole Cohort *N* = 225	Paediatric Nephrology *N* = 58	Adult & paediatrics *N* = 12
Role distribution	Doctor: 50% (*n* = 112) Scientist: 18.7% (*n* = 42) AHP: 31.5% (*n* = 71)	Doctor: 53.4% (*n* = 31) Scientist: 17.2% (*n* = 10) AHP: 29.3% (*n* = 17)	Doctor: 66.7% (*n* = 8) Scientist: 16.7% (*n* = 2) AHP: 16.7% (*n* = 2)
Top 3 geographical areas worked in	London: 20% (*n* = 45) North West: 16.4% (*n* = 37) South West and Scotland joint third 10.7% (*n* = 24)	North West: 31% (*n* = 18) London: 25.9% (*n* = 15) West Midlands: 22.4%, (*n* = 13)	North East: 25% (*n* = 3) South West: 16.2% (*n* = 2) 8.3% (*n* = 1) in each of North West, South East, Yorkshire, London, East Midlands, Wales, Northern Ireland
Healthcare setting	Secondary care: 75% (*n* = 169) Academia: 50% (*n* = 113) Research delivery: 16% (*n* = 38) Public Health: 3.5% (*n* = 8) Primary/community care: 3.1% (*n* = 7) Health and justice 0.8% (*n* = 2)	Secondary care: 82.7% (*n* = 48) Academia: 36.2% (*n* = 21) Research delivery: 15.5% (*n* = 9) Public health: 1.7% (*n* = 1)	Secondary care: 83.3% (*n* = 10) Research delivery: 100% (*n* = 12) Academia: 100% (*n* = 12)
Highest qualification	PhD: 52%, *n* = 118) Masters: 25% (*n* = 57) Undergraduate: 19% (*n* = 42) Diploma/certificate: 4% (*n* = 8)	PhD: 37.9% (*n* = 22) Masters: 20.7% (*n* = 12) Undergraduate: 36.2% (*n* = 21) Diploma/certificate: 5% (*n* = 3)	PhD: 91.7% (*n* = 11) MSc: 8.3% (*n* = 1)
Years experience	> 20 years: 42% (*n* = 94) 10–20 years: 28.4% (*n* = 64) 5–10 years: 19.1% (*n* = 43) < 5 years: 10.7% (*n* = 24)	> 20 years: 40% (*n* = 23) 10–20 years 22.4% (*n* = 13) 5–10 years: 19% (*n* = 11) < 5 years: 19% (*n* = 11)	> 20 years: 58.3% (*n* = 7) 10–20 years: 33.3% (*n* = 4) 5–10 years: 0 < 5 years: 8.3% (*n* = 1)
Time spent on research	> 75% of time: 25% (*n* = 56) 50–75%: 12.4% (*n* = 28) 25–50%: 13.3% (*n* = 30) 10–25%: 14.7% (*n* = 33) < 10%: 22.7% (*n* = 51) None: 12% (*n* = 27)	> 75% of time: 20.7% (*n* = 12) 50–75%: 8.6% (*n* = 5) 25–50%: 10.3% (*n* = 6) 10–25%: 15.5% (*n* = 9) < 10%: 29.3% (*n* = 17) None: 15.5% (*n* = 9)	> 75% of time: 16.7% (*n* = 2) 50–75%: 50% (*n* = 6) 25–50%: 16.7% (*n* = 2) 10–25%: 0 < 10%: 16.7% (*n* = 2) None: 0
Formal recognition of research	> 75% of time: 23.4% (*n* = 53) 50–75%: 10.8% (*n* = 24) 25–50%: 13.1% (*n* = 29) 10–25%: 6.3% (*n* = 14) < 10%: 8.6% (*n* = 19) None: 37.8% (*n* = 85)	> 75% of time: 17.2% (*n* = 10) 50–75%: 10.3% (*n* = 6) 25–50%: 7% (*n* = 4) 10–25%: 3% (*n* = 2) < 10%: 17% (*n* = 10) None: 43% (*n* = 25)	> 75% of time: 25% (*n* = 3) 50–75%: 25% (*n* = 3) 25–50%: 25% (*n* = 3) 10–25%: 8.3% (*n* = 1) < 10%: 0 None: 16.7% (*n* = 2)
Engagement (top 3)	Inform clinical practice: 68.9% (*n* = 155) QI/audit: 62.7% (*n* = 141) Collaborate/co-applicant for trials 53.3% (*n* = 120) Peer review journals/conference abstracts: 53.3% (*n* = 120)	Inform clinical practice: 69% (*n* = 40) Clinical audit/QI: 69% (*n* = 40) have Co-authored a research paper in the last 12 months: 56.9% (*n* = 33)	Clinical audit/QI: 75% (*n* = 9) Collaborate/co-applicant for research studies: 75% (*n* = 9) Signpost patients to studies: 66.7% (*n* = 8) Expert advisor: 66.7% (*n* = 8) Develop and lead research studies/act as Chief: 66.7% (*n* = 8)
Top 3 barriers	Lack of time: 64.4% (*n* = 145) Lack of funding: 60% (*n* = 135) Other roles taking priority: 53.3% (*n* = 120)	Lack of time: 60.3% (*n* = 35), Other roles taking priority: 53.4%, (*n* = 31) Lack of funds: 46.6% (*n* = 27).	Lack of funds 66.7% (*n* = 8) Lack of time: 50% (*n* = 6) Other roles take priority: 50% (*n* = 6) Desire for work/life balance: 50% (*n* = 6)
Top 3 motivators	Desire to improve patient care: 86.7%(*n* = 195) Increased job satisfaction: 78.2% (*n* = 176) To develop skills: 68% (*n* = 153)	Desire to improve patient care: 86% (*n* = 50) Increased job satisfaction: 77% (*n* = 45) To develop skills: 71% (*n* = 41)	Increased job satisfaction: 83% (*n* = 10) Desire to improve patient care: 83% (*n* = 10) To develop skills: 66.7% (*n* = 8) Desire to prove theory/hunch: 66.7% (*n* = 8)

### Barriers and motivators to engaging with research

The most commonly reported barriers to research engagement were lack of dedicated time (64.4%, *n* = 145), lack of funding (60%, *n* = 135), and competing professional roles (53.3%, *n* = 120) (Table 1). These barriers were consistent across professional groups, with only 2.7% (*n* = 6) reporting no barriers. Work–life balance and personal commitments were also frequently cited. Institutional barriers included limited availability of clinical academic and tenure-track posts, lack of coordinated research career pathways, and challenges associated with rotational training posts. Respondents highlighted difficulties balancing clinical and research responsibilities, limited recognition of research activity within job plans, and bureaucratic requirements related to ethics and governance. Limited access to funding opportunities, managerial support, and mentorship were commonly reported, alongside uncertainty about accessing support and perceived gaps in research skills. Feelings of intimidation and fear of making mistakes were noted, particularly among allied health professionals. Additional concerns included restricted fellowship support, lack of diversity and inclusion, and insufficient training opportunities.

The main motivators for contributing to research included the desire to improve patient care (86.7%, *n* = 195), increased job satisfaction (78.2%, *n* = 176) and to develop professional skills (68%, *n* = 153) (Table 1). More scientists also reported being motivated by career advancement and wanting to make a scientific impact. Two people specifically commented that they liked the increased flexibility with research, whilst two enjoyed being able to supervise and train the next generation of younger researchers.

### Academic skills of the workforce

**Fig. 2:**
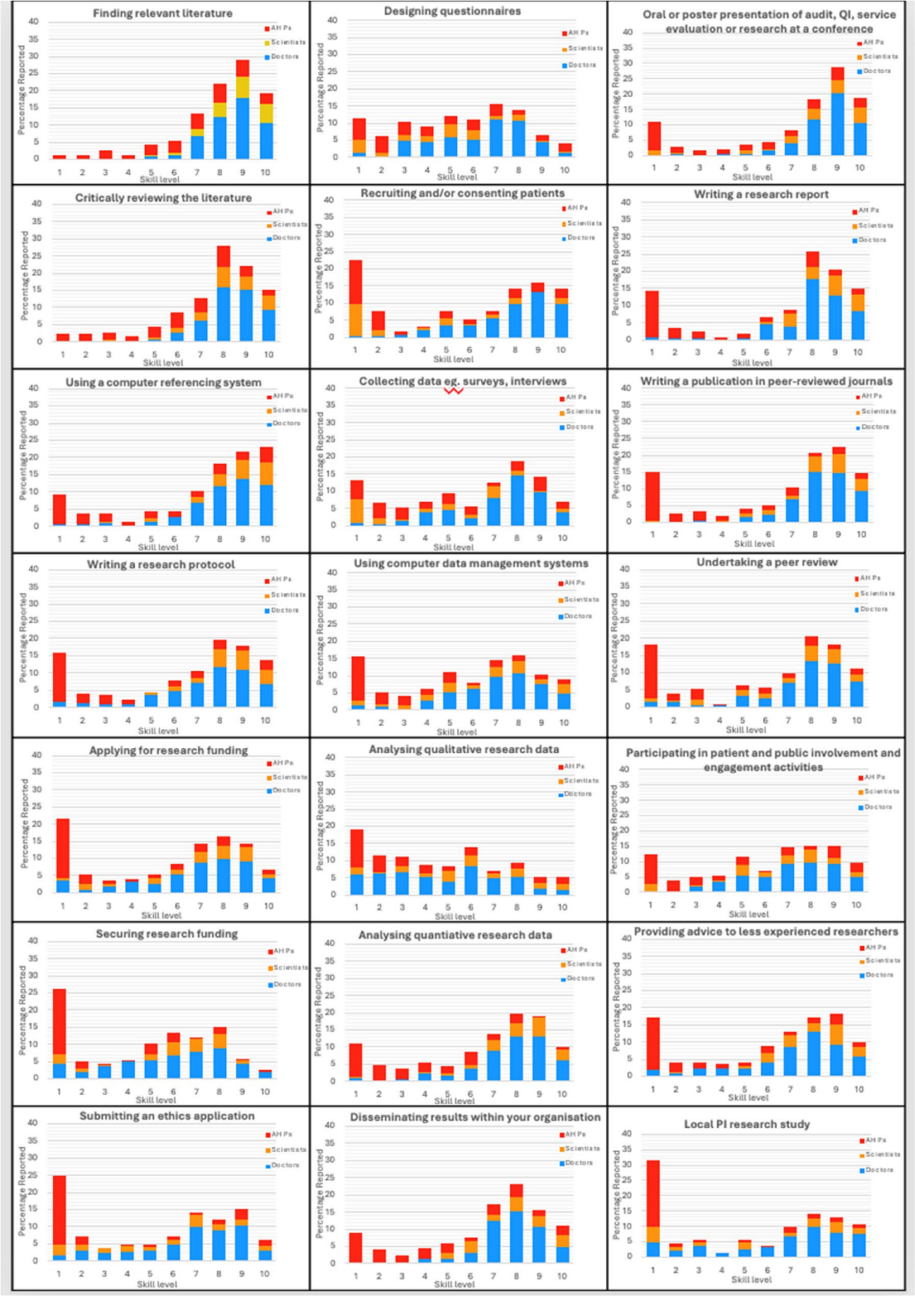
Variation in Self-Reported Research Skill Confidence Among Allied Health Professionals (AHP), Scientists, and Medical Doctors. Self-reported confidence levels for a range of core research skills are shown, according to the respondent’s profession where 0 represents least confidence and 10 represents greatest confidence

In terms of skills levels, respondents felt least confident in applying and securing research funding, submitting ethical approval applications, recruiting and/or consenting patients and being a local PI for research studies (Fig. 2). Generally, the cohort reported more confidence in finding relevant literature and performing critical analysis, using computer referencing, and writing research protocols when compared to writing research reports and presenting the results at conferences. AHPs were generally less confident in all research skill categories when compared to doctors and scientists. Scientists were generally less confident with designing questionnaires, recruiting, and consenting patients and collecting data, whereas the skill mix amongst doctors was very varied.

## Discussion

In the current era of a kidney failure crisis causing pressure on high-cost services, establishing a secure academic workforce to drive forward healthcare advances in this high-demand clinical speciality is a vital component in the strategy to change the future for patients living with kidney disease. While workforce pressures in kidney services are substantial, research and clinical care should not be viewed as competing priorities. Research underpins advances in prevention, treatment, and service delivery that reduce long-term demand on high-cost services. Research-active organisations consistently demonstrate better patient outcomes and greater system efficiency. Clinical academic roles embed research within frontline care and facilitate rapid translation of evidence into practice, rather than reducing clinical capacity. Reducing research activity to expand short-term workforce numbers risks limiting future improvements and entrenching existing inefficiencies. A balanced approach that strengthens both clinical and research capacity is therefore essential to improving patient outcomes and service sustainability.

The aim of this project was therefore to conduct a national approach to determining improvements for the academic workforce specialising in nephrology, determining their engagement in research along with their skillsets, perceived barriers, and motivators to research. The findings presented in this document will be used to inform a strategy to secure a skilled research workforce using an integrated approach with adult and paediatric clinical academic colleagues working in partnership, together with valued colleagues such as basic scientists and AHPs.

Concerningly, the age range of participants in this cohort reflected previous data showing an aging workforce in clinical academia [5]. With 30.4% of respondents over the age of 50 years, 70% with at least 10 years of experience and over 50% with a PhD qualification, and just 8.4% of respondents were under the age of 30 years; this suggests the current workforce may not be sustainable. Attempts to attract younger clinicians to academia have been in place however, the number of resident doctors interested in a clinical academic careers unfortunately declines over time after qualification [14]. A previous study by Bottomley et al. [12] showed that amongst resident doctors working in nephrology, 35% were training for a PhD and 50% were involved in research [12]. In the US, research is a mandatory part of nephrology training to encourage academia, however most colleagues ultimately spend the majority of their time working clinically once training is completed [15]. This pattern is also evident in the UK, where an estimated four clinical PhD trainees are needed to generate a single senior clinical academic, with lack of sustained funding commonly cited as a major obstacle to career advancement [7, 11]. The Royal College of Physicians reported in 2022 that 57% of its members wanted to be more involved in research, but noted that little progress had been made in actively removing the barriers to participation [16]. There is a hint of optimism however, as within the next age category of 31–40 years of age, 28.6% of respondents fell within group, and given long training times, this group may provide a more meaningful indicator of future workforce capacity. This suggests a substantial cohort are progressing through early- to mid-career stages who may form the next generation of senior clinical academics and research leaders. This supports a degree of workforce succession potential.

In this current study, time was the most widely reported barrier to engaging with research. Although 81% of respondents had research included in their job description, 37.8% did not have time formally recognised in their job plan, despite engaging in research being a requirement for Doctors and for advanced nurse practitioners by national colleges [17, 18]. Research is also a fundamental component of advanced-level pharmacy practice in the UK, as advocated by the Royal Pharmaceutical Society [19]. Many healthcare institutions have research as a priority objective and widely report the benefits of research, including increased patient confidence in staff, reduced mortality and increased quality of healthcare [20, 21].The need for a work-life balance was also reported as a barrier in this study, as research often needs to be conducted in a clinician’s own time. As the financially restricted environment is likely to continue, normalising a culture of research and incorporating learning into clinical care will reduce the dedicated time needed to actively engage. Furthermore, ensuring that clinicians spend time doing work they find meaningful can prevent burnout [22], whereas allocated time for clinicians to conduct research has been recognised to promote workforce retention [23]. The lack of university tenured posts was frequently reported as a challenge by both scientists and doctors. Whilst some barriers can be managed in the speciality, the major, recurring barriers need to be addressed in a national approach beyond Nephrology.

The high proportion of respondents (111/225) spending less than 25% of their time on research raises important questions about workforce capacity. However, clinicians who engage in research, even for a limited proportion of their time, play a key role in generating clinically relevant questions, supporting recruitment, and facilitating translation into practice. Their contribution therefore complements that of full-time PhD researchers. We believe that the limited research time reported by many respondents is likely to reflect structural constraints, including service pressures and limited access to protected academic time, rather than lack of interest or capability. An effective research workforce requires a balanced model that supports both dedicated researchers and clinician scientists.

Funding was cited as the second most common barrier in this study, in line with previous reports [7, 11, 12, 24] and the under recognition of the impact of kidney disease on society may be a contributory factor. Interestingly, despite the vast experience of the respondents, there was a low confidence reported in completing funding applications. Awareness of funding options was mainly reported as a barrier by AHPs and this group were least confident in all aspects of research skills, as mirrored in previous reports [25]. We acknowledge that self-reported skill level and confidence are subjective and were not independently verified. As such, reported gaps may reflect perceived confidence rather than actual competence or research success. Consequently, initiatives to support improvement may be most effectively targeted at increasing confidence, familiarity with research processes, and access to mentoring, alongside the development of technical skills.

Previous literature, has shown that AHPs can feel overwhelmed when transitioning to academia [9]. The under-representation of AHPs in research has prompted targeted efforts to address this imbalance, however with few role models or mentors within their field, as shown by the very low proportion of AHPs in clinical academia in our study, it may be difficult to access the routes into academia. Whilst mentorship may be a solution to overcome some of the barriers, that have subtle differences across the professions, this is challenging in small, underrepresented groups such as academic colleagues in paediatric nephrology or AHPs. The positive confidence in analysing basic literature allows a strategy that doesn’t need to dedicate efforts to address this need.

Paediatric nephrology research presents distinct scientific, ethical, and operational challenges, including smaller patient populations, greater regulatory requirements, and the need for age-specific methodologies. These factors can limit research activity and capacity compared with adult services. As a result, targeted recruitment and support of PhD- and MD-trained researchers within paediatric nephrology may be particularly important to sustain research output and innovation in this field.

The findings from the present study provide a useful comparison with Bottomley et al. [12], however, notable differences exist in respondent demographics [12]. The former survey specifically focused on early-career practitioners and trainees, whereas the present data set includes a significantly higher proportion of senior, experienced professionals, such as professors and principal investigators, who also possess higher overall academic qualifications. This demographic difference likely explains the higher reported research engagement in the current survey cohort (81.4%) compared to the 72%, despite a similar lack of formally recognized time [12]. Motivations also differed. The present data strongly indicates that improving patient care and clinical services are the primary motivators, while the *Bottomley et al.* noted a link between engagement and external factors like increased patient satisfaction. Furthermore, while the Bottomley et al. cohort desired mentorship as a strategy for engagement, a lack of mentorship was cited as a barrier in the current data set (20.9%), indicating a universal need for support across all career stages. Despite a four-year gap between the data collections, key issues remain persistent challenges in the field, including: (i) lack of time and funding as top barriers; (ii) mentorship as a critical enabler; and (iii) skills deficits related to grant applications and administrative complexity, particularly for less experienced individuals.

Whilst this study aimed to capture an overview of the academic status of the current nephrology workforce in 2025, its limitations must be highlighted. The survey was sent out via UK Kidney Association and Kidney Research UK e-news for those working over 50% of their time in nephrology, and participation was optional, therefore it is unknown if this captured a representative cohort of professionals working in nephrology. Questionnaires may be inherently biased, as those who are the most engaged in research will be most likely to respond. Self-reporting of skills was also subjective. It should also be noted that many clinicians enter academia at a later stage in their careers therefore the age range may not be truly reflective of the incoming workforce and specific work with the younger generation is therefore required. Despite these limitations, this project has obtained a wealth of data to support improvements.

## Conclusions

Overall, this study has demonstrated a potential ageing nephrology academic workforce. Despite a wide motivation for research, the barriers of time, funding and balancing clinical roles remain. A dedicated national workforce strategy and integrating research into clinical practice to facilitate a culture change may be the most promising solution.

A study of this nature should not merely be observational but should lead to a call to action. We have provided a short and longer term list of actions (see Table 2). The role of a committed national body such as the UK Kidney Association will be fundamental to achieve this goals as well as national funding agencies and charities that support kidney research.

**Table 2:** Short- and longer-term actions based on this survey

Action Area	Details / Roles
Short-Term Actions (1–3 years)	
1. Integrate Research into Clinical Practice	• UK Kidney Association (UKKA) can work with NHS Trusts to embed research into clinical job plans and highlight models of good practice through guidance and training events. • Funding agencies such as Kidney Research UK could pilot schemes where clinicians are given protected research sessions aligned with patient care pathways.
2. Address Funding Barriers and Skills Gaps	• UKKA could commission targeted training programmes in grant writing and research leadership for early career nephrology researchers. • Kidney Research UK could expand awareness campaigns about small-grant opportunities, especially for AHPs and paediatric nephrology researchers. • UKKA could coordinate national webinars and mentorship networks focused on funding applications.
3. Strengthen Early Career Pathways	• UKKA could establish a mentorship scheme pairing senior academic nephrologists with early career clinicians and AHPs. • Kidney Research UK / LifeArc / Wellcome / NIHR could provide pump-priming fellowships for junior clinicians to gain protected time to start research projects. • MRC / UKRI could embed academic exposure into nephrology training through joint clinical–academic training awards.
Long-Term Actions (3 + years)	
4. Develop a National Workforce Strategy	• UKKA, in collaboration with UKRI, MRC, and Kidney Research UK, could lead the development of a national nephrology academic workforce framework to address ageing demographics, retention, and succession planning. • MRC and UKRI could ensure nephrology is prioritised in future strategic funding calls, recognising the scale of the kidney failure crisis.
5. Foster Collaborative and Inclusive Networks	• UKKA could convene multi-disciplinary research consortia linking adult and paediatric nephrology, basic scientists, and AHPs, with funding agencies providing infrastructure support. • Kidney Research UK could fund national networking and leadership programmes for under-represented groups (e.g., AHPs, paediatric nephrology academics). • MRC / UKRI could promote large-scale interdisciplinary programmes in kidney research, creating a culture where research participation is the norm across the workforce.

## Supplementary Information

Supplementary Material 1.

## Data Availability

No datasets were generated or analysed during the current study.

## Abbreviations

ACF: Academic Clinical Fellowship
AHP: Allied Health Professional
CKD: Chronic Kidney Disease
GMC: General Medical Council
HEI: Higher Education Institution
MSc: Master of Science
NIHR: National Institute for Health and Care Research
PI: Principal Investigator
PhD: Doctor of Philosophy
RCP: Royal College of Physicians
RCN: Royal College of Nursing
RPS: Royal Pharmaceutical Society
UK: United Kingdom
UKKA: UK Kidney Association
UKRI: UK Research and Innovation
