# Supplementary material for: The UK kidney association 2025 academic census: a national survey to identify workforce improvements
Source: BMC Med Educ. 2026 Feb 20;26:499. doi: 10.1186/s12909-026-08855-y (PMC13032373; doi:10.1186/s12909-026-08855-y)
Supplement: Supplementary file 1 — Supplementary Material 1. [file 12909_2026_8855_MOESM1_ESM.pdf]

The UKKA 2025 Academic Census

The UKKA are developing a comprehensive research strategy. To do this, we are need to understand the current research environment so that it can better support and advocate for renal research.

This anonymous survey that will take 2 minutes to complete, is intended for any health care professional, clinician scientists and/or basic scientists who spend the majority (>50%) of their working time in the kidney field. We aim to capture all aspects of research from basic science to applied health research, to provide details of current kidney research capacity.

The following questions will help us to review the makeup of our community and ensure that everyone is represented.

Thank you in advance for taking the time to complete the below questions.

* Indicates required question

1. What is your current age? (optional)

Mark only one oval.

- <25
- 25-30
- 31-40
- 41-50
- 51-60
- 60+

2. What is your ethnicity? (optional)

Ethnic origin is not about nationality, place of birth or citizenship. It is about the group to which you perceive you belong. Please tick the below appropriate box. If 'Other', please state in box below.

Dropdown

Mark only one oval.

- Asian British
- Indian
- Pakistani
- Bangladeshi
- Chinese
- Black British
- African
- Caribbean
- White and Black Caribbean
- White and Black African
- White and Asian
- British/English/Welsh/Scottish/Northern Irish
- Irish
- Gypsy or Irish Traveller
- Arab
- Other

3. Other Ethnicity Group

4. What is your professional background? Select all that apply. *

Mark only one oval.

- Nurse - Research Delivery
- Nurse - Patient Facing
- Physio
- Pharmacist
- OT
- Doctor - International Medical Graduate
- Doctor - EEA Medial Graduate
- Doctor - UK Medical Graduate
- Psychologist
- Social Worker
- Dietitian
- Technician
- Scientist - post doctoral
- Scientist - pre doctoral
- Scientist - PI
- Other: __________

5. What is your job title? *

____

6. Where are you based? *

Mark only one oval.

- Scotland
- North East England
- North West England
- Central England
- South East England
- South West England
- London
- Wales
- Northern Ireland
- Other: ______________________

7. Who is your current employer(s)? *

____

____

____

____

____

8. Which clinical centre are you affiliated with? *

____

____

____

____

____

9. Which academic centre are you affiliated with? (if applicable) *

10. What setting do you work in? Tick all that apply. *

Tick all that apply.

- [ ] Primary Care - General Practice
- [ ] Primary Care - Primary Care Network
- [ ] Primary Care - Community Services
- [ ] Primary Care - Integrated Care Board (e.g. medicines optimisation team)
- [ ] Secondary Care - Acute Trust
- [ ] Secondary Care - Specialist Trust
- [ ] Research Delivery
- [ ] Academia
- [ ] Health and Justice
- [ ] Public Health
- [ ] Multi-sector
- [ ] Other: ______________________

11. Which nephrology setting(s) do you work within? Tick all that apply. *

Tick all that apply.

- [ ] Dialysis Centre
- [ ] Paediatric Dialysis Centre
- [ ] Acute Nephrology Ward
- [ ] Acute Paediatric Nephrology Ward
- [ ] Chronic Nephrology Ward
- [ ] Chronic Paediatric Nephrology Ward
- [ ] Renal Transplant Ward
- [ ] Paediatric Renal Transplant Ward
- [ ] Nephrology OPD
- [ ] Paediatric Nephrology OPD
- [ ] MDT Clinic (adults)
- [ ] MDT Clinic (paeds)
- [ ] MDT Clinic (mixed)
- [ ] Renal Dietetics
- [ ] Paediatric Renal Dietetics
- [ ] Renal Pharmacy
- [ ] Paediatric Renal Pharmacy
- [ ] Renal Physio
- [ ] Paediatric Renal Physio
- [ ] Renal Occupational Therapy
- [ ] Paediatric Renal Occupational Therapy
- [ ] Other: ____________________

12. How many years of professional experience have you accumulated? *

Mark only one oval.

- ( ) <5 years
- ( ) 5-10 years
- ( ) 10-20 years
- ( ) 20+ years

13. What is your highest level of qualification? *

Mark only one oval.

- Certificate/Diploma
- Degree
- MSc/Post-Graduate
- PhD
- Other: ____________________

14. Are research related activities part of your current role description or have they been in previous roles? *

Mark only one oval.

- Current
- Former
- Current and Former
- Not applicable

15. How much of your time in your current role is spent on research or research-related activity? *

Mark only one oval.

- None of my time (0%)
- More than 0% but less than 10% of my time
- More than 10% but less than 25% of my time
- More than 25% but less than 50% of my time
- More than 50% but less than 75% of my time
- More than 75% of my time

16. How much of your time in your current role is formally recognised for research or research-related activity? *

Mark only one oval.

- None of my time (0%)
- More than 0% but less than 10% of my time
- More than 10% but less than 25% of my time
- More than 25% but less than 50% of my time
- More than 50% but less than 75% of my time
- More than 75% of my time

17. Is research engagement/activity discussed as part of your annual appraisal? *

Mark only one oval.

- Yes, routinely
- Only if I bring it up / when I am currently involved in research
- Never

18. At what level do you currently engage in research? Tick all that apply. *

Tick all that apply.

- [ ] I don't currently use/engage in research at all
- [ ] I use research evidence to inform my clinical practice
- [ ] I am involved in clinical audit/quality improvement research activity to evaluate and/or improve clinical services
- [ ] I raise awareness/signpost patients to research studies in my area
- [ ] I support clinical trials/research through screening/recruitment/treatment delivery
- [ ] I act as an expert advisor/sit on steering groups or research studies
- [ ] I am a collaborate/co-applicant for research studies/trials
- [ ] I take on the role of Site Principal Investigator
- [ ] I develop and lead research studies/trials and/or act as Chief Investigator
- [ ] I am employed as a researcher at a University
- [ ] I undertake research as a university Principal Investigator
- [ ] I undertake research as part of a university team under a Principal Investigator
- [ ] I take part in/run a journal club
- [ ] I peer review journal articles/conference abstracts
- [ ] Other: ____________________

19. Please indicate if you have completed any of the following research activities in the past 12 months. Tick all that apply. *

Tick all that apply.

- [ ] Co authored a research-based paper for publication
- [ ] Patient and public involvement and engagement activities
- [ ] Presented research findings at a conference
- [ ] No research activity completed in the past 12 months
- [ ] Secured local research funding as Principle Investigator
- [ ] Secured local research funding as Co-Principle Investigator
- [ ] Secured national research funding as Principle Investigator
- [ ] Secured national research funding as Co-Principle Investigator
- [ ] Secured international research funding as Principle Investigator
- [ ] Secured international research funding as Co-Principle Investigator
- [ ] Other: ____________________

Please rate your own current success or skill level for each of the following aspects, relevant to you, by selecting a score on a 1-10 scale, where 1 = no success/skill (complete novice) and 10 = highest possible success/skill (expert).

20. Finding relevant literature *

Mark only one oval.

1	2	3	4	5	6	7	8	9	10
◯	◯	◯	◯	◯	◯	◯	◯	◯	◯

21. Critically reviewing the literature *

Mark only one oval.

1	2	3	4	5	6	7	8	9	10
◯	◯	◯	◯	◯	◯	◯	◯	◯	◯

22. Using a computer referencing system (e.g. Endnote) *

Mark only one oval.

1	2	3	4	5	6	7	8	9	10
◯	◯	◯	◯	◯	◯	◯	◯	◯	◯

23. Writing a research protocol *

Mark only one oval.

1	2	3	4	5	6	7	8	9	10
◯	◯	◯	◯	◯	◯	◯	◯	◯	◯

24. Applying for research funding *

Mark only one oval.

1	2	3	4	5	6	7	8	9	10
◯	◯	◯	◯	◯	◯	◯	◯	◯	◯

25. Securing research funding *

Mark only one oval.

1	2	3	4	5	6	7	8	9	10
◯	◯	◯	◯	◯	◯	◯	◯	◯	◯

26. Submitting an ethics application *

Mark only one oval.

1	2	3	4	5	6	7	8	9	10
◯	◯	◯	◯	◯	◯	◯	◯	◯	◯

27. Designing questionnaires *

Mark only one oval.

1	2	3	4	5	6	7	8	9	10
◯	◯	◯	◯	◯	◯	◯	◯	◯	◯

28. Recruiting and/or consenting patients *

Mark only one oval.

1	2	3	4	5	6	7	8	9	10
◯	◯	◯	◯	◯	◯	◯	◯	◯	◯

29. Collecting data e.g. surveys, interviews *

Mark only one oval.

1	2	3	4	5	6	7	8	9	10
◯	◯	◯	◯	◯	◯	◯	◯	◯	◯

30. Using computer data managements systems *

Mark only one oval.

1	2	3	4	5	6	7	8	9	10
◯	◯	◯	◯	◯	◯	◯	◯	◯	◯

31. Analysing qualitative research data *

Mark only one oval.

1	2	3	4	5	6	7	8	9	10
◯	◯	◯	◯	◯	◯	◯	◯	◯	◯

32. Analysing quantitative research data *

Mark only one oval.

1	2	3	4	5	6	7	8	9	10
◯	◯	◯	◯	◯	◯	◯	◯	◯	◯

33. Disseminating results within your organisation *

Mark only one oval.

1	2	3	4	5	6	7	8	9	10
◯	◯	◯	◯	◯	◯	◯	◯	◯	◯

34. Oral or poster presentation of audit, quality improvement, service evaluation or research at a conference *

Mark only one oval.

1	2	3	4	5	6	7	8	9	10
◯	◯	◯	◯	◯	◯	◯	◯	◯	◯

35. Writing a research report *

Mark only one oval.

1	2	3	4	5	6	7	8	9	10
◯	◯	◯	◯	◯	◯	◯	◯	◯	◯

36. Writing for publication in peer-reviewed journals *

Mark only one oval.

1	2	3	4	5	6	7	8	9	10
⬭	⬭	⬭	⬭	⬭	⬭	⬭	⬭	⬭	⬭

37. Undertaking peer review *

Mark only one oval.

1	2	3	4	5	6	7	8	9	10
⬭	⬭	⬭	⬭	⬭	⬭	⬭	⬭	⬭	⬭

38. Participating in Patient and Public involvement and engagement activities *

Mark only one oval.

1	2	3	4	5	6	7	8	9	10
⬭	⬭	⬭	⬭	⬭	⬭	⬭	⬭	⬭	⬭

39. Providing advice to less experienced researchers *

Mark only one oval.

1	2	3	4	5	6	7	8	9	10
⬭	⬭	⬭	⬭	⬭	⬭	⬭	⬭	⬭	⬭

40. Local Principal Investigator (PI) research study *

Mark only one oval.

1	2	3	4	5	6	7	8	9	10
◯	◯	◯	◯	◯	◯	◯	◯	◯	◯

41. What are the barriers to research for you personally? Tick all that apply. *

Tick all that apply.

- [ ] Issues with visa
- [ ] Lack of time for research
- [ ] Lack of suitable backfill
- [ ] Rotational post
- [ ] Other work roles take priority
- [ ] Lack of funds for research
- [ ] Lack of awareness of funding opportunities
- [ ] Lack of support from management
- [ ] Lack of mentor/support
- [ ] Unsure how to access support/networks
- [ ] Lack of access to equipment for research
- [ ] Lack of software for research
- [ ] Not interested in research
- [ ] Other personal commitments
- [ ] Desire for work/life balance
- [ ] Lack of a co-ordinated approach to research
- [ ] Lack of skills for research
- [ ] Intimidated by research language
- [ ] Limited by fear of getting it wrong
- [ ] Lack of diversity and inclusion in research
- [ ] Lack of reward in relation to career progression
- [ ] Lack of clinical academic positions
- [ ] Lack of support for applying for Fellowships
- [ ] Lack of University tenured posts
- [ ] Other: ____________

42. Rank the top three personal barriers from your above selections. *

____

43. What are the motivators to do research for you personally? Tick all that apply. *

Tick all that apply.

- [ ] To develop skills
- [ ] Career advancement
- [ ] Increased job satisfaction
- [ ] Desire to improve patient care or service delivery
- [ ] Study or research scholarships available
- [ ] Dedicated time for research
- [ ] Research written into role description
- [ ] Colleagues doing research
- [ ] Mentors available to supervise
- [ ] Research encouraged by managers
- [ ] Grant funds
- [ ] Links to universities
- [ ] Forms part of Post-Graduate study
- [ ] Opportunities to participate at own level
- [ ] Problem identified that needs changing
- [ ] Desire to prove a theory/hunch
- [ ] To keep the brain stimulated
- [ ] Increased credibility
- [ ] Desire to improve sustainability (e.g. environmental, societal and economic sustainability)
- [ ] To have a scientific impact
- [ ] Other: ________________

44. If you consider yourself to be a senior academic, would you be willing to offer mentorship? If so, please include your email address below.

____

45. Which of the following does your organisation do to support research? Tick all that apply. *

Tick all that apply.

- [ ] Don't know
- [ ] Has role models and named research leads
- [ ] Identifies and celebrates research successes
- [ ] Provides support for developing research submissions
- [ ] Has adequate resources to support staff research training
- [ ] Has funds/equipment/admin to support research activities
- [ ] Has a plan or policy for research development
- [ ] Has senior managers that support research
- [ ] Ensures staff career pathways are available in research
- [ ] Ensures organisation planning is guided by evidence
- [ ] Has patients and public involved in research
- [ ] Accesses external funding for research
- [ ] Promotes clinical practice based on evidence
- [ ] Encourages research activities relevant to practice
- [ ] Has software programmes for analysing research data
- [ ] Has mechanisms to monitor research quality
- [ ] Has identified experts accessible for research advice
- [ ] Supports a multi-disciplinary approach to research
- [ ] Has regular forums/bulletins to present research findings
- [ ] Engages external partners (e.g. universities) in research
- [ ] Supports applications for research scholarships/degrees
- [ ] Supports the peer-reviewed publications of research

46. Would you like to be contacted with the summary results (META DATA) of this survey? If so, please include your email address below. *

____

Thank you for taking the time to complete the census.

Please click submit below.

This content is neither created nor endorsed by Google.

Google Forms
